# Bioactive Phenolic and Isocoumarin Glycosides from the Stems of *Homalium paniculiflorum*

**DOI:** 10.3390/molecules23020472

**Published:** 2018-02-22

**Authors:** Shou-Yuan Wu, Yan-Hui Fu, Qi Zhou, Meng Bai, Guang-Ying Chen, Si-Yu Zhao, Chang-Ri Han, Xiao-Ping Song

**Affiliations:** 1 Key Laboratory of Tropical Medicinal Plant Chemistry of Ministry of Education, Hainan Normal University, Haikou 571158, China; wushouyuan2012@163.com (S.-Y.W.); fuyanhui80@163.com (Y.-H.F.); zhouqi0313@163.com (Q.Z.); XXbai2014@163.com (M.B.); chgying123@163.com (G.-Y.C.); 13876740482@163.com (S.-Y.Z.); 2 Key Laboratory of Medicinal and Edible Plants Resources of Hainan Province, Hainan Institute of Science and Technology, HaiKou 571126, China

**Keywords:** *Homalium paniculiflorum*, phenolic glycosides, isocoumarin glycosides, NO production inhibition

## Abstract

Two new phenolic glycosides (**1** and **2**) and two new isocoumarin glycosides (**3** and **4**), along with 14 known compounds (**5**–**18**), were isolated from the stems of *Homalium paniculiflorum*. Their structures were established on the basis of extensive spectroscopic analyses and chemical methods. All new compounds were evaluated for their anti-inflammatory activities via examining the inhibitory activity on nitric oxide (NO) production induced by lipopolysaccharide (LPS) in mouse macrophage RAW 264.7 cells in vitro. Compounds **1** and **4** exhibited inhibitory activities with IC_50_ values of 30.23 ± 1.23 μM and 19.36 ± 0.19 μM, respectively.

## 1. Introduction

**Figure 1:**
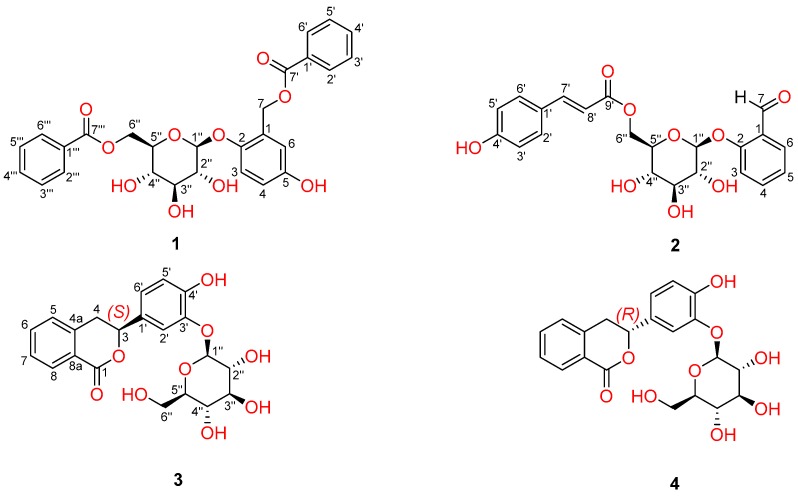
Chemical structures of compounds **1**–**4**.

The genus *Homalium* (Flacourtiaceae), comprising about 130 species, are mainly distributed in temperate and subtropical regions. There are about 12 species of this genus in China, growing from the southwest to Taiwan. Among which, *H. paniculiflorum* is a Chinese endemic plant, only distributed in China’s Hainan Island [1]. Previous phytochemical investigations on the genus have afforded various types of compounds including phenolic glycosides, xanthenes, iridoids, coumarins, triterpenoids and alkaloids, which showed a wide variety of interesting bioactivities including anti-bacterial, anti-oxidant, anti-viral, anti-plasmodial, hypolipidemic and hypoglycemic activities [2,3,4,5,6,7,8,9,10,11]. As a Chinese endemic medicinal plant, up to now, there is only a preliminary investigation on the chemical composition of *H. paniculiflorum* performed by us recently [10,11]. As a part of our ongoing research into structurally and biologically interesting natural products from tropical medicinal plants in China, a further chemical investigation on *H. paniculiflorum* was undertaken and had led to the isolation of two new phenolic glycosides and two new isocoumarin glycosides (**1**–**4**) (Figure 1), along with 14 known compounds, vanillin (**5**) [12], 2,4-dihydroxybenzaldehyde (**6**) [13], 4-hydroxy-3,5-dimethoxybenzaldehyde (**7**) [14], *β*-hydroxypropiovanillone (**8**) [15], coniferaldehyde (**9**) [16], 2-hydroxy phenyl-*β*-glucoside (**10**) [17], helicin (**11**) [18], salirepin (**12**) [4], salireposide (**13**) [3], homaloside B (**14**) [3], poliothrysoside (**15**) [19], itoside H (**16**) [20], 3-phenylisocoumarin (**17**) [7] and 3-(3′-hydroxyphenyl) isocoumarin (**18**) [8]. The structures of the new compounds **1**–**4** were elucidated by extensive spectroscopic analyses and chemical methods, while the known compounds were identified by comparisons their data with those reported in the literature. In addition, all new compounds were evaluated for their anti-inflammatory activities via examining the inhibitory activity on NO production induced by LPS in mouse macrophage RAW 264.7 cells in vitro. Compounds **1** and **4** exhibited inhibitory effects with IC_50_ values comparable to that of L-NMMA (NG-Methyl-l-arginine). Herein, we describe the isolation, structural elucidation and anti-inflammatory activities of these new compounds.

## 2. Results and Discussion

The methanol extract of the stems of *H. paniculiflorum* was suspended in water and extracted successively with petroleum ether and EtOAc. The EtOAc extract was repeatedly subjected to silica gel CC, reversed-phase C_18_ silica gel CC, Sephadex LH-20 CC and semi-preparative HPLC, to yield 18 compounds, including two new phenolic glycosides (**1** and **2**) and two new isocoumarin glycosides (**3** and **4**), as shown in Figure 1.

**Figure 2:**
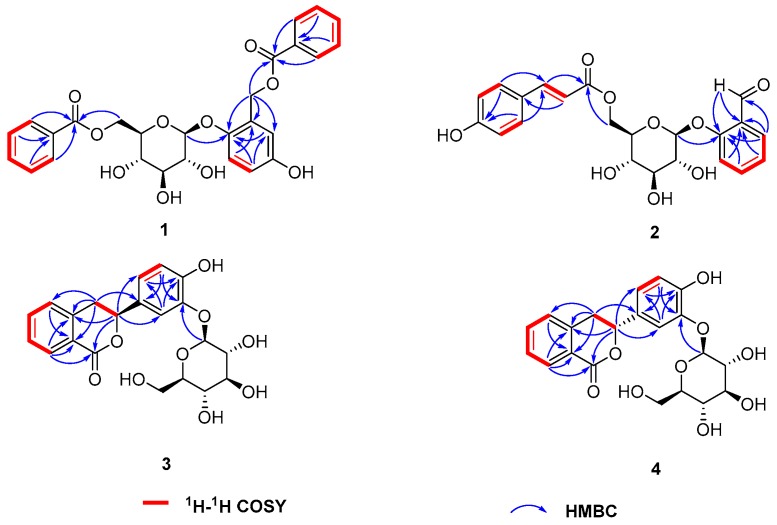
Key HMBC and ^1^H-^1^H COSY correlations for compounds **1**–**4**.

Compound **1** was obtained as a white amorphous powder. Its molecular formula was determined as C_27_H_26_O_10_ by HRESIMS (*m*/*z* 533.1413 [M + Na]^+^, calcd. 533.1418), indicating 15 degrees of unsaturation. Its IR spectrum showed the presence of hydroxyl groups (3468 cm^−1^), an ester carbonyl group (1708 and 1676 cm^−1^) and phenyl groups (1620, 1518 and 1493 cm^−1^). The UV maxima at 262 and 218 nm indicated that **1** possessed aromatic rings. The ^13^C-NMR and DEPT data (Table 1) revealed the presence of 27 carbon atoms, including 20 sp^2^ carbon atoms, five sp^3^ methines and two sp^3^ methylenes, which were attributable to two benzoate groups, one benzyl alcohol group and one glucopyranosyl moiety. The above data revealed that the structure of **1** was similar to that of itoside H (**16**) [20], except that the hydroxyl group at C-2′ was substituted by a hydrogen atom, which was supported by the HMBC correlations of H-2′ to C-4′ (*δ*_C_ 134.2), C-6′ (*δ*_C_ 130.6) and C-7′ (*δ*_C_ 167.8), as well as the ^1^H-^1^H COSY correlations from H-2′ to H-6′. Detailed analysis of 2D-NMR (HSQC, HMBC and ^1^H-^1^H COSY) spectra confirmed the structure of **1** (see Figure 2). Furthermore, the coupling constant of the anomeric proton resonating at *δ*_H_ 4.79 (1H, d, *J* = 7.6 Hz, H-1″) suggested that the glucopyranosyl moiety was *β*-glucoside. In order to further confirm the structure of **1**, the acid hydrolysis reaction of **1** was carried out. As a result, a *β*-d-glucose was produced as the sole sugar identified on the basis of the same *R_f_* value on co-TLC and the almost identical optical value by comparing with that of an authentic sugar sample. Therefore, compound **1** was determined as 4-hydroxy-2-{[(benzoyl)oxy] methyl}phenyl-*β*-d-glucopyranoside-6-benzoate, as shown in Figure 1. 

Compound **2** was obtained as a white amorphous powder. Its molecular formula was determined as C_22_H_22_O_9_ by HRESIMS with *m*/*z* 453.1163 [M + Na]^+^ (calcd. for C_22_H_22_O_9_Na, 453.1162), indicating 12 degrees of unsaturation. The IR spectrum showed the presence of hydroxyl group (3442 cm^−1^), carbonyl groups (1720 and 1675 cm^−1^) and phenyl groups (1612, 1516 and 1468 cm^−1^) functionalities. In the ^1^H-NMR spectrum of **2** (see Figure S6 in Supplementary Material), two groups of mutual coupled deshielded protons at *δ*_H_ 7.31 (1H, d, *J* = 7.6 Hz, H-3), 7.50 (1H, dd, *J* = 7.6, 7.6 Hz, H-4), 7.08 (1H, dd, *J* = 7.6, 7.6 Hz, H-5) and 7.76 (1H, d, *J* = 7.6 Hz, H-6), as well as 7.46 (2H, d, *J* = 8.4 Hz, H-2′, H-6′) and 6.82 (2H, d, *J* = 8.4 Hz, H-3′, H-5′) suggested the presence of one 1,2-disubstituted benzene ring and one 1,4-disubstituted benzene ring, respectively. The ^13^C-NMR and DEPT data (Table 1) revealed the existence of 22 carbon atoms, including 16 sp^2^ carbon atoms, five sp^3^ methines and one sp^3^ methylenes, which were attributable to one *p*-coumaroyl moiety, one benzaldehyde moiety and one glucopyranosyl moiety. The above data revealed that the structure of **1** was similar to that of 6′-*O*-(*Z*)-*p*-coumaroylsalicin [21]. Further comparisons of ^1^H-NMR, ^13^C-NMR and DEPT data of **2** with 6′-*O*-(*Z*)-*p*-coumaroylsalicin indicated that there were two major differences between their structures. Firstly, the hydroxymethyl group at C-1 in 6′-*O*-(*Z*)-*p*-coumaroylsalicin was substituted by an aldehyde group in **2**, which was supported by the HMBC correlations of H-6 to C-7 (*δ*_C_ 191.8), as well as H-7 to C-1 (*δ*_C_ 127.5), C-2 (*δ*_C_ 161.3) and C-6 (*δ*_C_ 128.7). Secondly, the orientation of the olefinic bond between C-7′ and C-8′ was assigned as *E*, based on the typical coupling constant between H-7′ and H-8′ (*J* = 16.0 Hz). Detailed analysis of 2D-NMR (HSQC, HMBC and ^1^H-^1^H COSY) spectra confirmed the planar structure of **2**, as shown in Figure 2. Furthermore, the coupling constant of the anomeric proton resonating at 5.05 (1H, d, *J* = 7.6 Hz, H-1″) suggested that the glucopyranosyl moiety was *β*-glucoside. In order to further confirm the structure of **2**, the acid hydrolysis reaction of **2** was carried out. As a result, a *β*-d-glucose was produced as the sole sugar identified on the basis of the same *R_f_* value on co-TLC and the almost identical optical value by comparing with that of an authentic sugar sample. Thus, compound **2** was established as 6-*O*-(*E*)-*p*-coumaroyl-*β*-d-glucopyranoside-2-benzaldehyde, as shown in Figure 1.

Compound **3** was isolated as a white amorphous powder, possessing the molecular formula of C_21_H_22_O_9_ as established by HRESIMS (*m*/*z* 441.1158 [M + Na]^+^; calcd. for C_21_H_22_O_9_Na, 441.1162) with an index of hydrogen deficiency of **11**. In ^1^H-NMR spectrum of **3**, two groups of mutual coupled deshielded protons at *δ*_H_ 7.45 (1H, d, *J* = 7.6 Hz, H-5), 7.48 (1H, dd, *J* = 7.6, 7.6 Hz, H-7), 7.65 (1H, dd, *J* = 7.6, 7.6 Hz, H-6) and 7.99 (1H, d, *J* = 7.6 Hz, H-8), as well as 6.72 (1H, dd, *J* = 8.2, 2.0 Hz, H-6′), 6.92 (1H, d, *J* = 2.0 Hz, H-2′) and 7.02 (1H, d, *J* = 8.2 Hz, H-5′) suggested the presence of one 1,2-disubstituted benzene ring and one 1,2,4-trisubstituted benzene ring, respectively. The ^13^C-NMR and DEPT data (Table 2) revealed the presence of 21 carbon atoms, including 13 sp^2^ carbon atoms, six sp^3^ methines and two sp^3^ methylenes, which were attributable to one dihydroisocoumarin skeleton and one glucopyranosyl moiety. The above data revealed that the structure of **3** was similar to that of thunberginol G 3′-*O*-glucoside [22], except that the hydroxyl group at C-8 was substituted by a hydrogen atom, which was supported by the HMBC correlations of H-8 to C-1 (*δ*_C_ 164.9), C-4a (*δ*_C_ 140.0) and C-8a (*δ*_C_ 124.8), as well as the ^1^H-^1^H COSY correlations from H-5 to H-8. Detailed analysis of 2D-NMR (HSQC, HMBC and ^1^H-^1^H COSY) spectra confirmed the planar structure of **3** (see Figure 2). Furthermore, the coupling constant of the anomeric proton resonating at 4.59 (1H, d, *J* = 7.6 Hz, H-1″) suggested that the glucopyranosyl moiety was *β*-glucoside. In order to further confirm the structure of **3**, the acid hydrolysis reaction of **3** was carried out. As a result, a *β*-d-glucose was produced as the sole sugar identified on the basis of the same *R_f_* value on co-TLC and the almost identical optical value by comparing with that of an authentic sugar sample. In addition, the absolute configuration of the aglycone of **3**, only holding one chiral center at C-3, was determined as *S*, based on its specific optical rotation of ${\left[\alpha \right]}_{D}^{24}$ −153.0 (*c* 0.9, CH_3_OH), which was similar with that of (*S*)-3,4-dihydro-3-phenylisochromen-1-one (${\left[\alpha \right]}_{D}^{24}$ −158.0), whose structure and absolute configuration had been determined by a combination of spectroscopic analyses and chemical methods [23]. Hence, compound **3** was determined as 3*S*-(4′-hydroxyl-3′-*O*-*β*-d-glucopyranosyl phenyl)-dihydroiso coumarin, as shown in Figure 1.

The molecular formula of **4** was established as C_21_H_22_O_9_ by HRESIMS (*m*/*z* 441.1161, [M + Na]^+^; calcd. for C_21_H_22_O_9_Na, 441.1162), the same with that of **3**. The ^1^H and ^13^C-NMR data (Table 2) of **3** were nearly identical to those of **4**. Detailed analysis of 2D-NMR (HSQC, HMBC and ^1^H-^1^H COSY) spectra confirmed that **4** shared the same planar structure with **3** (see Figure 2). The specific rotation of **4**, ${\left[\alpha \right]}_{D}^{24}$ +187.0 (*c* 0.10, CH_3_OH), suggested that its configuration should be different from that of **3** (${\left[\alpha \right]}_{D}^{24}$ −89.2). In order to further confirm the structure of **4**, the acid hydrolysis reaction of **4** was carried out. As a result, a *β*-d-glucose was produced as the sole sugar identified on the basis of the same *R_f_* value on co-TLC and the almost identical optical value by comparing with that of an authentic sugar sample. In addition, the absolute configuration of the aglycone of **4** was determined as *R*, in consideration of its converse optical rotation of ${\left[\alpha \right]}_{D}^{24}$ +146.0 (*c* 0.9, CH_3_OH) with that of the aglycone of **3**, which was very similar with that of (*R*)-3,4-dihydro-3-phenylisochromen-1-one (${\left[\alpha \right]}_{D}^{24}$ +168.5), whose structure and absolute configuration had been determined by a combination of spectroscopic analyses and chemical methods [24]. Accordingly, compound **4** was identified as the 3-epimer of **3**, namely, 3*R*-(4′-hydroxyl-3′-*O*-*β*-d-glucopyranosylphenyl)-dihydro isocoumarin, as shown in Figure 1.

All new compounds were evaluated for their anti-inflammatory properties via examining the inhibitory activity on NO production induced by LPS in mouse macrophage RAW 264.7 cells in vitro. As a result, new compounds **1** and **4** showed significant inhibitory activities with the IC_50_ values of 30.23 ± 1.23 μM and 19.36 ± 0.19 μM, respectively. While the positive control, l-NMMA (NG-Monomethyl-l-arginine), showed an inhibitory activity with the IC_50_ value at 32.88 ± 2.59 μM. The other compounds showed no inhibitory activity on NO production in this assay (IC_50_ > 100 μM). No cytotoxicities were observed in compounds **1**–**4** treated cells (cell viability > 90%).

The above findings may be used as an explanation of the folk use of *H. paniculiflorum*, which was used as an anti-inflammatory drug in China [1,11]. These findings also suggest that the phenolic glycoside and isocoumarin glycoside with significant inhibitory activities on NO production isolated from *H. paniculiflorum* could be used for the development of new anti-inflammatory agents. 

## 3. Experimental Section

### 3.1. General Experiment Procedure

Optical rotations were measured with a JASCO P-1020 digital polarimeter (JASCO Corporation, Tokyo, Japan). UV spectra were recorded on a Beckman DU 640 spectrophotometer (Beckman Instruments, Fullerton, CA, USA). IR spectra were obtained on a Nicolet 6700 spectrophotometer (Thermo Scientific, Madison, WI, USA). NMR spectra were run on a Bruker 400 MHz spectrometer (Bruker Biospin, Rheinstetten, Germany) using TMS as an internal standard. HRESIMS spectra were measured on a Q-TOF Ultima Global GAA076 LC mass spectrometer (Waters Corporation, Milford, MA, USA). Semi-preparative HPLC was performed on an Agilent 1260 LC series (Agilent Technologies, Santa Clara, CA, USA) with a DAD detector using an Agilent Eclipse XDB-C_18_ column (250 × 9.4 mm, 5 µm). Silica gel (Qing Dao Hai Yang Chemical Group Co., Qingdao, China; 200–300 mesh) and reversed-phase C_18_ silica gel (YMC; 50 μm) were used for column chromatography (CC). Pre-coated silica gel plates (Yan Tai Zi Fu Chemical Group Co., Yantai, China; G60, F-254) were used for thin layer chromatography (TLC). 

### 3.2. Plant Material

The stems of *H. paniculiflorum* were collected from Bawangling Nature Reserve, Hainan Province China, in August 2012 and identified by Prof. Qiong-Xin Zhong, College of Life Science, Hainan Normal University. A voucher specimen (No. SONG20120818) has been deposited at the Key Laboratory of Tropical Medicinal Plant Chemistry of Ministry of Education, Hainan Normal University. 

### 3.3. Extraction and Isolation

The powdered stems of *H. paniculiflorum* (22.0 kg) were refluxed with methanol for three times. The solvent was evaporated *in vacuum* to obtain a crude extract. After suspended in water, the crude extract was extracted successively with petroleum ether and EtOAc. The EtOAc extract (100.0 g) was subjected to silica gel CC, eluted with petroleum ether/EtOAc (from 1:0 to 0:1) yielding five fractions (Fr.1–Fr.5). Fr.4 (16.8 g) was subjected to reversed-phase C_18_ silica gel CC eluting with CH_3_OH/H_2_O (from 40% to 100%) to afford five fractions (Fr.4A–Fr.4F). Fraction 4A (1.8 g) was purified by Sephadex LH-20 CC eluted with CH_3_OH, then separated by a series of silica gel CC eluted with petroleum ether/EtOAc 5:5 to afford **7** (16.3 mg), **11** (29.7 mg), **12** (23.5 mg) and **14** (21.3 mg). Fraction 4B (2.3 g) was purified by Sephadex LH-20 CC eluted with CH_3_OH, then separated by a series of silica gel CC eluted with petroleum ether/acetone 6:4 to afford **1** (7.8 mg), **2** (9.2 mg) and **16** (35.8 mg). Fraction 4C (2.0 g) was purified using Sephadex LH-20 CC eluted with CH_3_OH, then separated by a silica gel CC eluted with petroleum ether/EtOAc 8:2 to yield **3** (8.6 mg), **4** (7.8 mg), **9** (40.2 mg) and **17** (11.6 mg). Fraction 4F (860 mg) was purified using Sephadex LH-20 CC eluted with CH_3_OH, then separated by a semi-preparative HPLC using an Agilent Eclipse XDB-C_18_ column with 75% CH_3_OH/H_2_O to afford compound **5** (10.2 mg), **6** (29.0 mg), **8** (22.8 mg), **10** (19.6 mg), **13** (12.6 mg), **15** (15.3 mg) and **18** (5.3 mg).

*4-Hydroxy-2-{[(benzoyl)oxy]methyl}phenyl-β-d-glucopyranoside-6-benzoate* (**1**): Colorless amorphous powder; ${\left[\alpha \right]}_{D}^{24}$ −24.8 (*c* 0.12, CH_3_OH); IR (KBr) *ν*_max_ 3468, 2973, 1708, 1676, 1620, 1518, 1493, 1431, 1302, 1211 and 1080 cm^−1^; UV (CH_3_OH) *λ*_max_ (log *ε*) 218 (3.03), 262 (4.06) and 303 (1.92); ^1^H and ^13^C-NMR data (Table 1); ESIMS *m*/*z* 533 [M + Na]^+^; HRESIMS *m*/*z* 533.1413 ([M + Na]^+^; calcd. for C_27_H_26_O_10_Na, 533.1418). 

*6-O-(E)-p-Coumaroyl-β-d-glucopyranoside-2-benzaldehyde* (**2**): Colorless amorphous powder; ${\left[\alpha \right]}_{D}^{24}$ +54.2 (*c* 0.14, CH_3_OH); IR (KBr) *ν*_max_ 3442, 1720, 1675, 1612, 1516, 1468, 1276, 1065 and 704 cm^−1^; UV (CH_3_OH) *λ*_max_ (log *ε*) 222 (3.76), 272 (4.49) and 308 (1.98) nm; ^1^H and ^13^C-NMR data (Table 1); ESIMS *m*/*z* 453 [M + Na]^+^; HRESIMS *m*/*z* 453.1163 ([M + Na]^+^; calcd. for C_22_H_22_O_9_Na, 453.1162).

*3S-(4′-Hydroxyl-3′-O-β-d-glucopyranosylphenyl)-dihydroisocoumarin* (**3**): White amorphous powder; ${\left[\alpha \right]}_{D}^{24}$ −89.2 (*c* 0.13, CH_3_OH); IR (KBr) *ν*_max_ 3185, 1618, 1510, 1469, 1387, 1125 and 704 cm^−1^; UV (CH_3_OH) *λ*_max_ (log *ε*) 220 (4.58), 254 (4.21), 278 (2.56) and 326 (2.19) nm; ^1^H and ^13^C-NMR data (Table 2); ESIMS *m*/*z* 441 [M + Na]^+^; HRESIMS *m*/*z* 441.1158 ([M + Na]^+^; calcd. for C_21_H_22_O_9_Na, 441.1162).

*3R-(4′-Hydroxyl-3′-O-β-d-glucopyranosylphenyl)-dihydroisocoumarin* (**4**): White amorphous powder; ${\left[\alpha \right]}_{D}^{24}$ +187.0 (*c* 0.10, CH_3_OH); IR (KBr) *ν*_max_ 3188, 1616, 1516, 1472, 1388, 1127 and 699 cm^−1^; UV (CH_3_OH) *λ*_max_ (log *ε*) 223 (4.62), 258 (4.28), 282 (2.62) and 329 (2.28) nm; ^1^H and ^13^C-NMR data (Table 2); ESIMS *m*/*z* 441 [M + Na]^+^; HRESIMS *m*/*z* 441.1161 ([M + Na]^+^; calcd. for C_21_H_22_O_9_Na, 441.1162).

### 3.4. Acid Hydrolysis of Compounds ***1***–***4***

Compounds **1**–**4** (each 1.0–2.0 mg) were refluxed with 2 mL of 1 N HCl for 1 h at 100 °C. The reaction mixtures were extracted with EtOAc and the aqueous phase was compared to an authentic sugar sample by co-TLC (CHCl_3_-CH_3_OH-H_2_O-AcHO, 13:3:3:1, *R_f_* 0.46 for glucose). The identification of *β*-d-glucose in each aqueous layer was realized by comparing the optical rotation of the liberated glucose with that of an authentic sample of *β*-d-glucose (${\left[\alpha \right]}_{D}^{24}$ +55.0).

### 3.5. Inhibitory Assay of NO Production

Murine macrophage cell line RAW264.7 was obtained from Cell Bank of Chinese Academy of Sciences. RAW264.7 cells were seeded in 96-well cell culture plates (1.5 × 10^5^ cells/well) and treated with serial dilutions of the compounds with a maximum concentration of 100 μM in triplicate, followed by stimulation with 1 μg/mL LPS (Sigma, St. Louis, MO, USA) for 18 h. NO production in the supernatant was assessed by Griess reagents (Reagent A & Reagent B, respectively, Sigma). The absorbance at 570 nm was measured with a microplate reader (Thermo, Waltham, MA, USA). N^G^-Methyl-l-arginine acetate salt (l-NMMA, Sigma, Hongkong, China), a well-known nitric oxide synthase (NOS) inhibitor, was used as a positive control [25]. The viability of RAW264.7 cells was evaluated by the MTS assay simultaneously to exclude the interference of the cytotoxicity of the test compounds [26].

## 4. Conclusions

Phytochemical investigation on the stems of *H. paniculiflorum* resulted in the isolation of two new phenolic glycosides (**1** and **2**) and two new isocoumarin glycosides (**3** and **4**), along with 14 known compounds (**5**–**18**). Their structures were established on the basis of extensive spectroscopic analyses and chemical methods. All new compounds were evaluated for their anti-inflammatory activities via examining the inhibitory activity on NO production induced by LPS in mouse macrophage RAW 264.7 cells in vitro. Compounds **1** and **4** exhibited significant inhibitory activities with IC_50_ values comparable to that of l-NMMA. 

## Figures and Tables

**Table 1:** ^1^H and ^13^C-NMR data of compounds **1** and **2** in CD_3_OD.

Position	Compound 1		Compound 2	
	*δ*_H_^a^	*δ*_C_^b^	*δ*_H_^a^	*δ*_C_^b^
1		129.4 s		127.5 s
2		149.7 s		161.3 s
3	7.07 (1H, d, 8.0)	119.9 d	7.31 (1H, d, 7.6)	118.2 d
4	6.55 (1H, dd, 8.0, 2.0)	116.4 d	7.50 (1H, dd, 7.6, 7.6)	137.1 d
5		154.3 s	7.08 (1H, dd, 7.6, 7.6)	123.8 d
6	6.83 (1H, d, 2.0)	116.2 d	7.76 (1H, d, 7.6)	128.7 d
7*α*	5.46 (1H, d, 13.2)	63.1 t	10.50 (1H, s)	191.8 d
7*β*	5.41 (1H, d, 13.2)			
1′		131.3 s		127.1 s
2′	7.98 (1H, d, 7.6)	130.6 d	7.46 (1H, d, 8.4)	131.2 d
3′	7.45 (1H, dd, 7.6, 7.6)	129.6 d	6.82 (1H, d, 8.4)	116.9 d
4′	7.59 (1H, dd, 7.6, 7.6)	134.2 d		161.5 s
5′	7.45 (1H, dd, 7.6, 7.6)	129.6 d	6.82 (1H, d, 8.4)	116.9 d
6′	7.98 (1H, d, 7.6)	130.6 d	7.46 (1H, d, 8.4)	131.2 d
7′		167.8 s	7.61 (1H, d, 16.0)	146.8 d
8′			6.33 (1H, d, 16.0)	114.9 d
9′′				168.9 s
1′′	4.79 (1H, d, 7.6)	104.7 d	5.05 (1H, d, 7.6)	102.7 d
2′′	3.53–3.49 (1H, m)	75.0 d	3.61–3.57 (1H, m)	74.8 d
3′′	3.49–3.46 (1H, m)	78.0 d	3.55–3.50 (1H, m)	77.9 d
4′′	3.45–3.40 (1H, m)	72.1 d	3.47–3.43 (1H, m)	71.7 d
5′′	3.71–3.67 (1H, m)	75.5 d	3.78–3.74 (1H, m)	75.8 d
6′′*α*	4.69 (1H, dd, 11.6, 1.6)	65.3 t	4.53 (1H, dd, 11.6, 1.6)	64.5 t
6′′*β*	4.40 (1H, dd, 11.6, 7.6)		4.39 (1H, dd, 11.6, 6.8)	
1′′′		131.5 s		
2′′′	8.02 (1H, d, 7.6)	130.6 d		
3′′′	7.48 (1H, dd, 7.6, 7.6)	129.6 d		
4′′′	7.61 (1H, dd, 7.6, 7.6)	134.3 d		
5′′′	7.48 (1H, dd, 7.6, 7.6)	129.6 d		
6′′′	8.02 (1H, d, 7.6)	130.6 d		
7′′′		168.0 s		

^a^ Measured at 400 MHz; ^b^ Measured at 100 MHz.

**Table 2:** ^1^H and ^13^C-NMR data of compounds **3** and **4** in DMSO-*d*_6_.

Position	Compound 3		Compound 4	
	*δ*_H_^a^	*δ*_C_^b^	*δ*_H_^a^	*δ*_C_^b^
1		164.9 s		164.9 s
2				
3	6.04 (1H, dd, 11.6, 3.2)	74.2 d	5.94 (1H, dd, 11.6, 3.2)	74.3 d
4*α*	3.27–3.32 (1H, m)	33.7 t	3.40–3.43 (1H, m)	33.2 t
4*β*	3.12–3.15 (1H, m)		3.15–3.17 (1H, m)	
4a		140.0 s		140.0 s
5	7.45 (1H, d, 7.6)	127.5 d	7.39 (1H, d, 7.6)	127.5 d
6	7.65 (1H, dd, 7.6, 7.6)	133.9 d	7.64 (1H, dd, 7.6, 7.6)	133.9 d
7	7.48 (1H, dd, 7.6, 7.6)	127.8 d	7.47 (1H, dd, 7.6, 7.6)	128.0 d
8	7.99 (1H, d, 7.6)	129.3 d	7.97 (1H, d, 7.6)	129.2 d
8a		124.8 s		124.7 s
1′		129.9 s		129.3 s
2′	6.92 (1H, d, 2.0)	112.8 d	6.92 (1H, d, 2.0)	112.7 d
3′		146.9 s		147.1 s
4′		152.7 s		152.5 s
5′	7.02 (1H, d, 8.2)	118.2 d	7.09 (1H, d, 8.2)	117.6 d
6′	6.72 (1H, dd, 8.2, 2.0)	115.7 d	6.70 (1H, dd, 8.2, 2.0)	115.5 d
1′′	4.59 (1H, d, 7.6)	103.5 d	4.65 (1H, d, 7.6)	102.6 d
2′′	3.15–3.13 (1H, m)	73.3 d	3.16–3.14 (1H, m)	73.4 d
3′′	3.22–3.19 (1H, m)	77.1 d	3.23–3.19 (1H, m)	77.1 d
4′′	3.10–3.05 (1H, m)	69.9 d	3.11–3.04 (1H, m)	69.8 d
5′′	3.26–3.23 (1H, m)	76.3 d	3.26–3.24 (1H, m)	76.6 d
6′′*α*	3.74 (1H, dd, 11.6, 5.2)	61.0 t	3.69 (1H, dd, 11.6, 5.2)	60.8 t
6′′*β*	3.43 (1H, dd, 11.6, 6.0)		3.47 (1H, dd, 11.6, 6.0)	
4′-OH	9.28 (1H, s)		9.27 (1H, s)	

^a^ Measured at 400 MHz; ^b^ Measured at 100 MHz.

## Supplementary Materials

1D and 2D NMR spectra of compounds **1**–**4** as Supplementary Materials are available online.
