# Supplementary material for: Bioactive Phenolic and Isocoumarin Glycosides from the Stems of Homalium paniculiflorum
Source: Molecules. 2018 Feb 22;23(2):472. doi: 10.3390/molecules23020472 (PMC6017599; doi:10.3390/molecules23020472)
Supplement: Supplementary file 1 [file molecules-23-00472-s001.pdf]

Supporting Information

Bioactive Phenolic and Isocoumarin Glycosides from the Stems of *Homalium paniculiflorum*

Shou-Yuan Wu [1,†], Yan-Hui Fu [1,†], Qi Zhou [1], Meng Bai [1], Guang-Ying Chen [1], Si-Yu Zhao [1], Chang-Ri Han [2,*] and Xiao-Ping Song [1,*]

[1] Key Laboratory of Tropical Medicinal Plant Chemistry of Ministry of Education, Hainan Normal University, Haikou 571158, China; wushouyuan2012@163.com (S.-Y.W.); fuyanhui80@163.com (Y-H.F.); zhouqi0313@163.com (Q.Z.); XXbai2014@163.com (M.B.); chgying123@163.com (G.-Y.C.); 13876740482@163.com (S.-Y.Z.); sxp628@126.com (X.-P.S.)

[2] Key Laboratory of Medicinal and Edible Plants Resources of Hainan Province, Hainan Institute of Science and Technology, HaiKou, 571126, China; hchr116@126.com (C.-R.H.)

[*] Correspondence: sxp628@126.com; hchr116@126.com; Tel./Fax: +86-898-65889422

[†] These authors contributed equally to this work.

Contents of Supporting Information

Figure S1. ^{1}H NMR spectrum of compound **1** in CD_3OD.

Figure S2. ^{13}C NMR spectrum of compound **1** in CD_3OD.

Figure S3. HSQC spectrum of compound **1** in CD_3OD.

Figure S4. HMBC spectrum of compound **1** in CD_3OD.

Figure S5. ^{1}H-^{1}H COSY spectrum of compound **1** in CD_3OD.

Figure S6. ^{1}H NMR spectrum of compound **2**-in CD_3OD.

Figure S7. ^{13}C NMR spectrum of compound **2** in CD_3OD.

Figure S8. HSQC spectrum of compound **2** in CD_3OD.

Figure S9. HMBC spectrum of compound **2** in CD_3OD.

Figure S10. ^{1}H-^{1}H COSY spectrum of compound **2** in CD_3OD.

Figure S11. ^{1}H NMR spectrum of compound **3** in DMSO-d_6.

Figure S12. ^{13}C NMR spectrum of compound **3** in DMSO-d_6.

Figure S13. HSQC spectrum of compound **3** in DMSO-d_6.

Figure S14. HMBC spectrum of compound **3** in DMSO-d_6.

Figure S15. ^{1}H-^{1}H COSY spectrum of compound **3** in DMSO-d_6.

Figure S16. ^{1}H NMR spectrum of compound **4** in DMSO-d_6.

Figure S17. ^{13}C NMR spectrum of compound **4** in DMSO-d_6.

Figure S18. HSQC spectrum of compounds **4** in DMSO-d_6.

Figure S19. HMBC spectrum of compound **4** in DMSO-d_6.

Figure S20. ^{1}H-^{1}H COSY spectrum of compound **4** in DMSO-d_6.

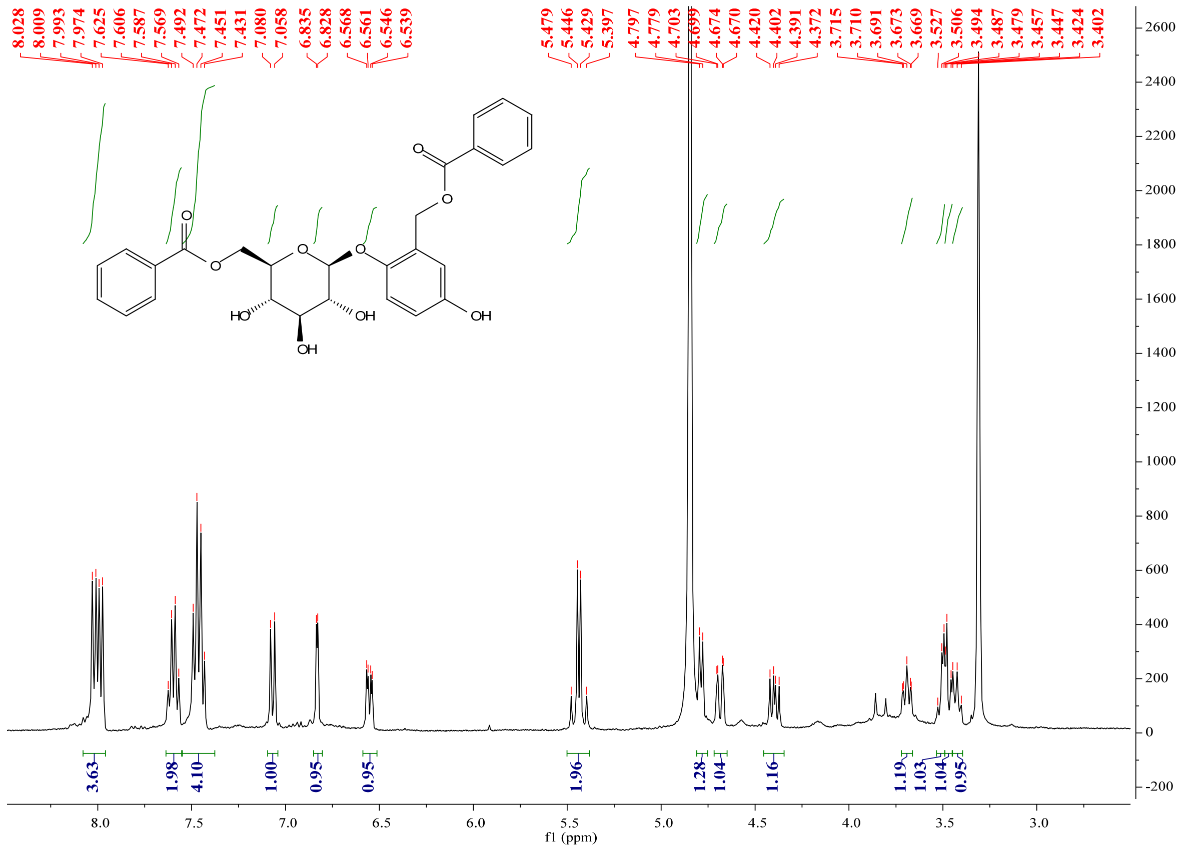

Figure S1. ^{1}H NMR spectrum of compound **1** in CD_3OD.

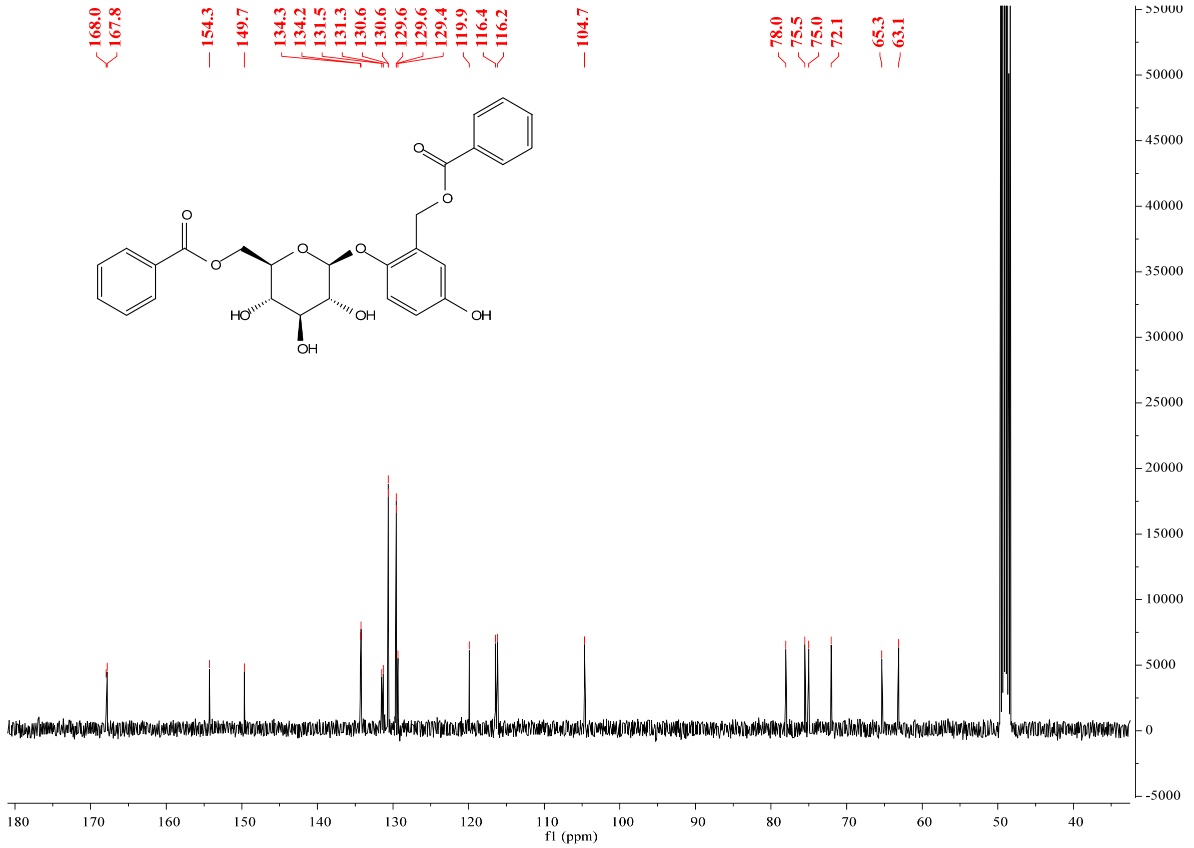

Figure S2. ^{13}C NMR spectrum of compound **1** in CD_3OD.

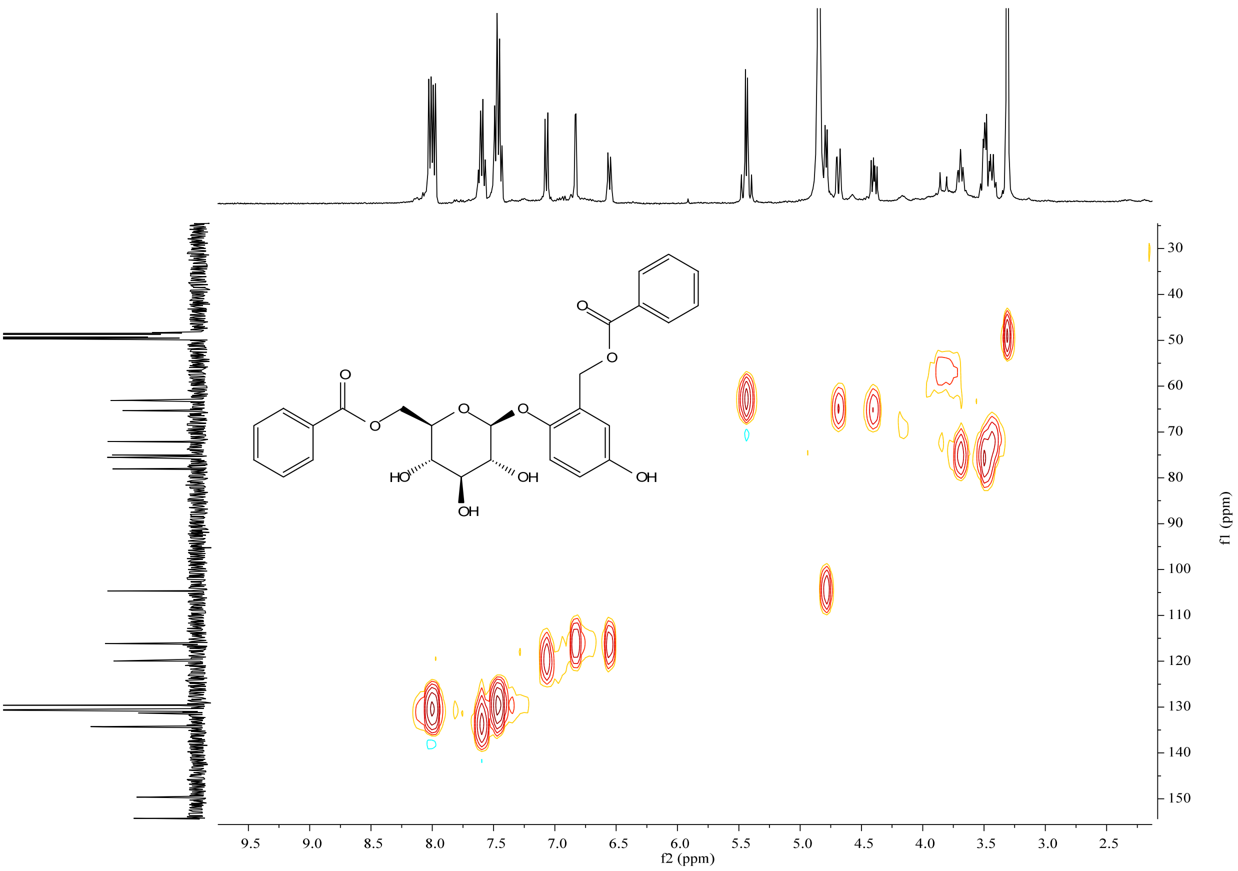

Figure S3. HSQC spectrum of compound **1** in CD_3OD.

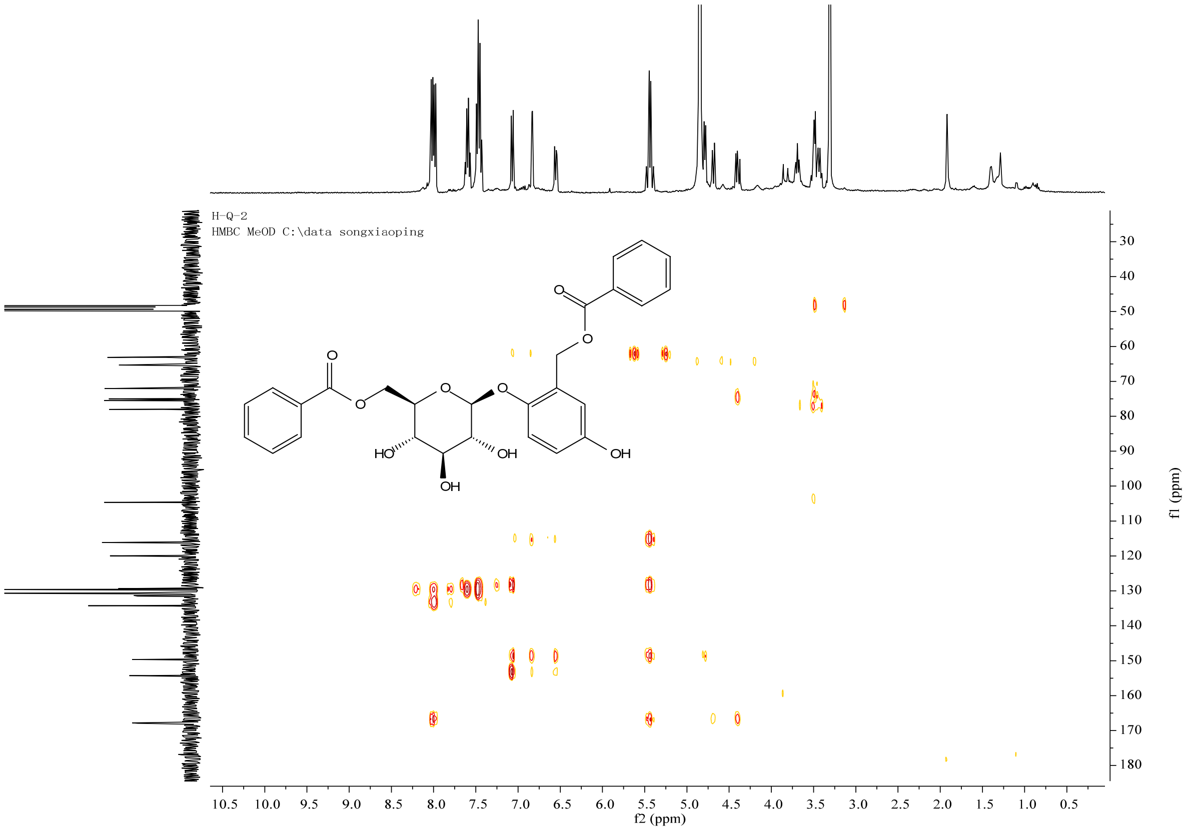

Figure S4. HMBC spectrum of compound **1** in CD_3OD.

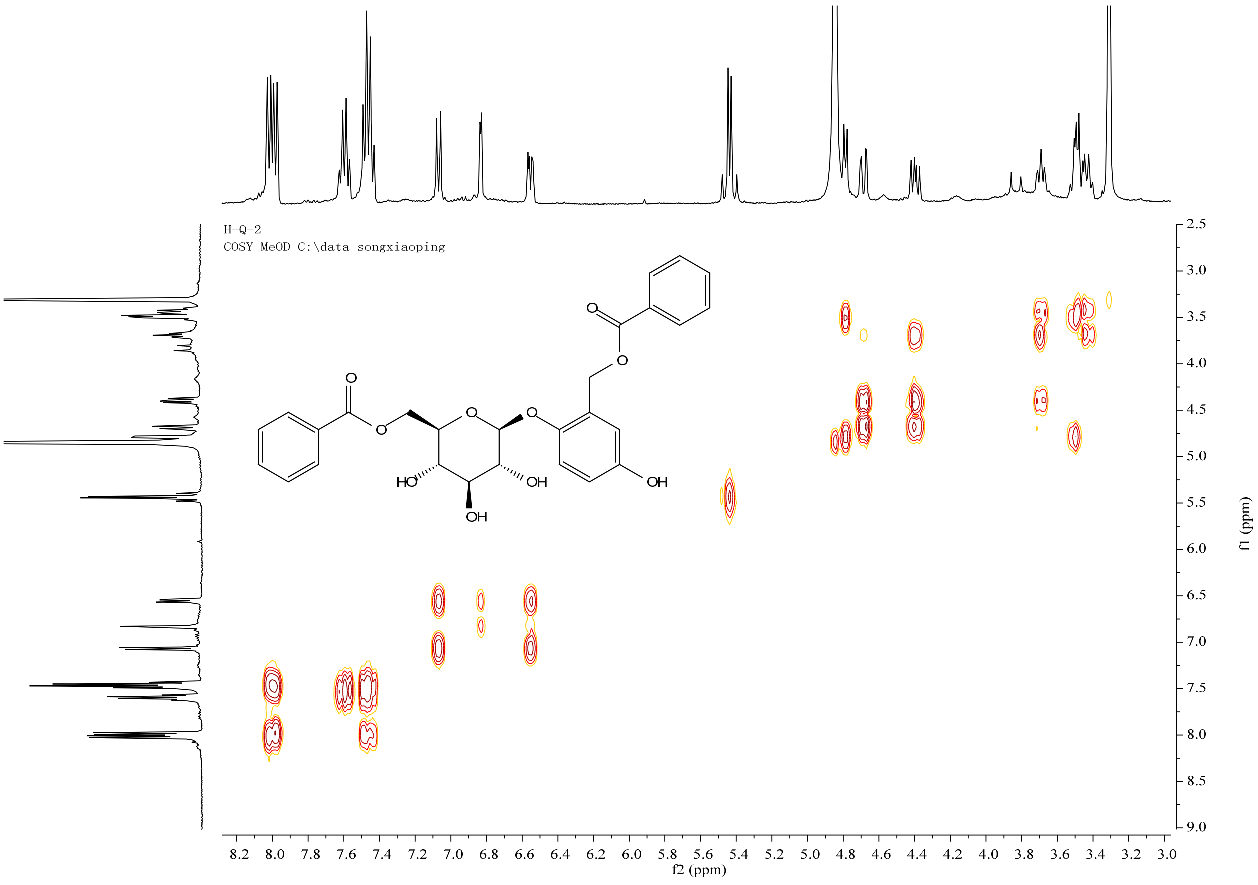

Figure S5. ^{1}H-^{1}H COSY spectrum of compound **1** in CD_3OD.

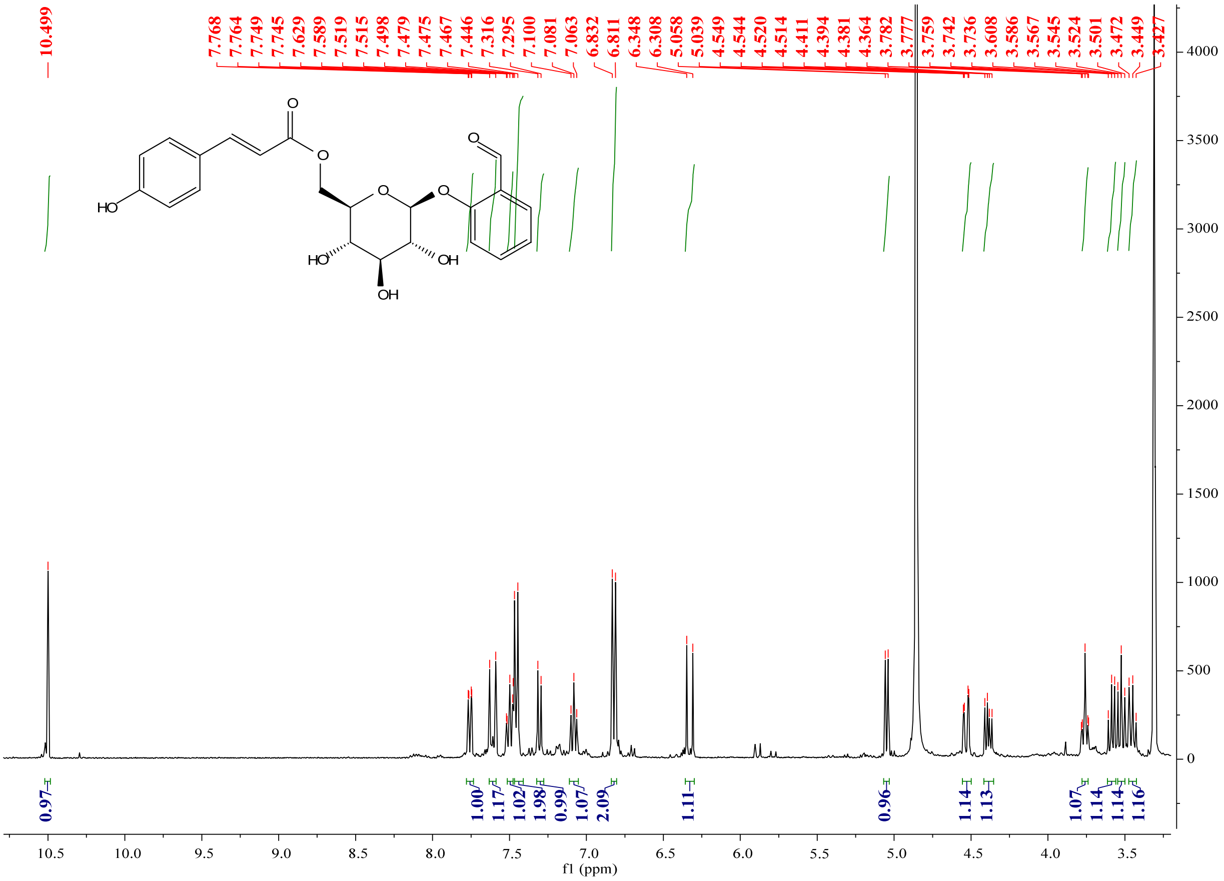

Figure S6. ^{1}H NMR spectrum of compound **2** in CD_3OD.

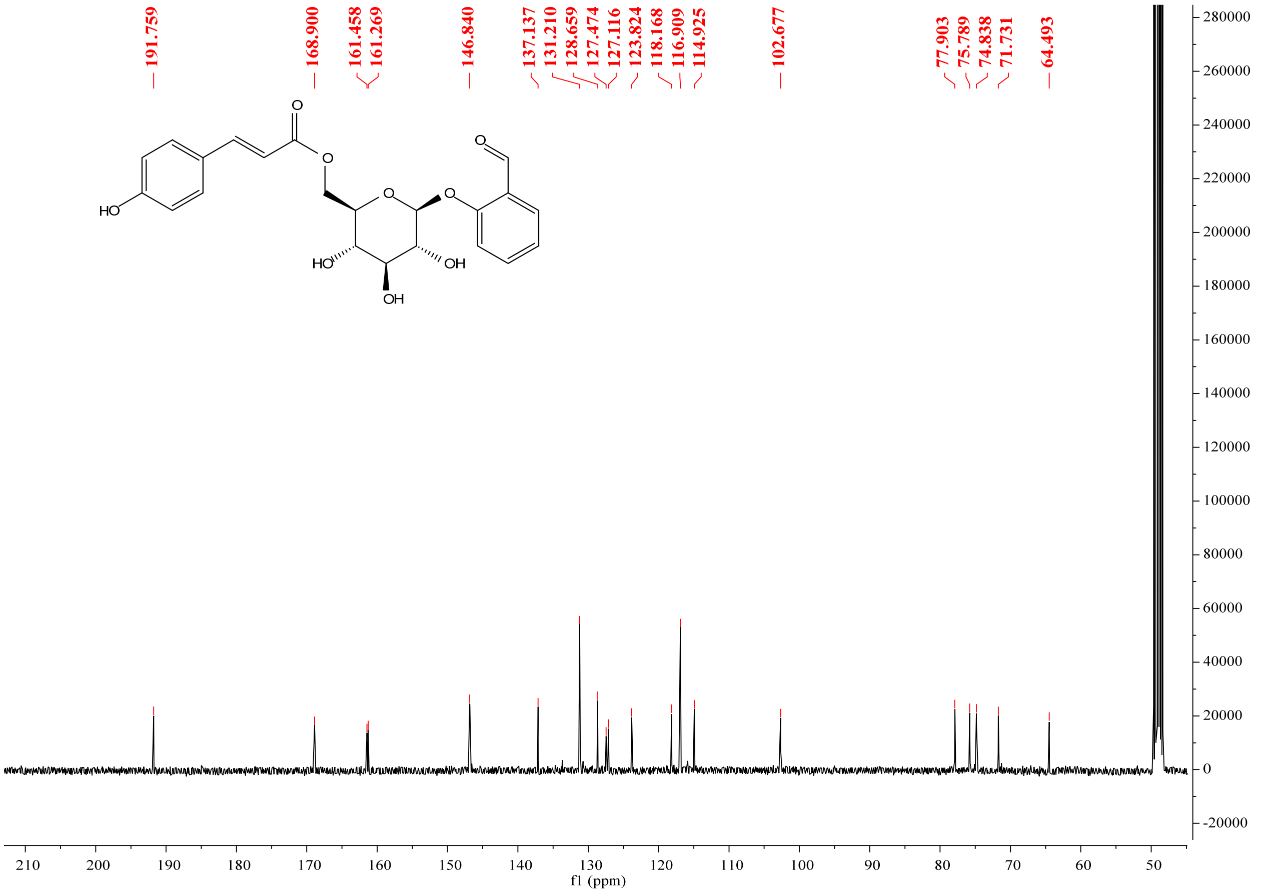

Figure S7. ^{13}C NMR spectrum of compound **2** in CD_3OD.

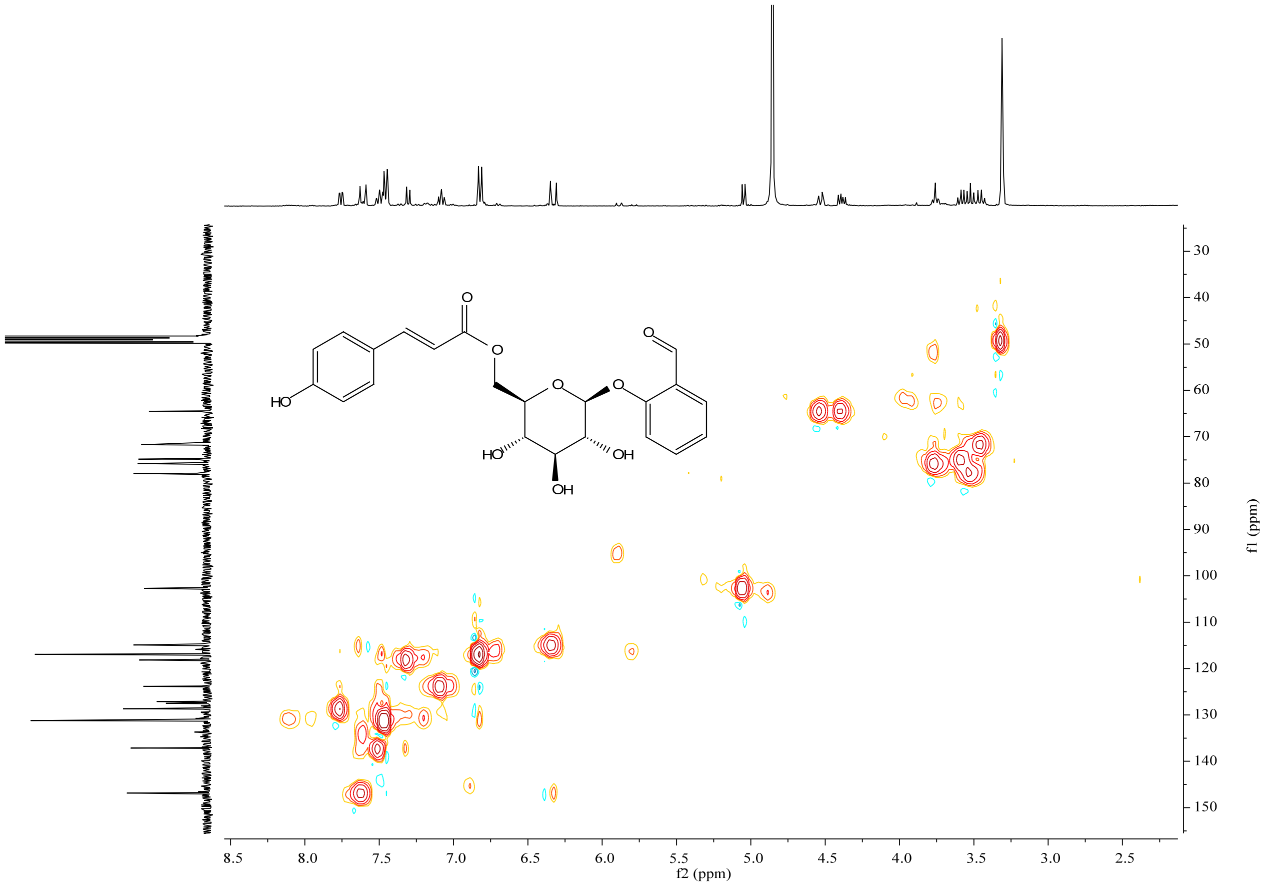

Figure S8. HSQC spectrum of compound **2** in CD_3OD.

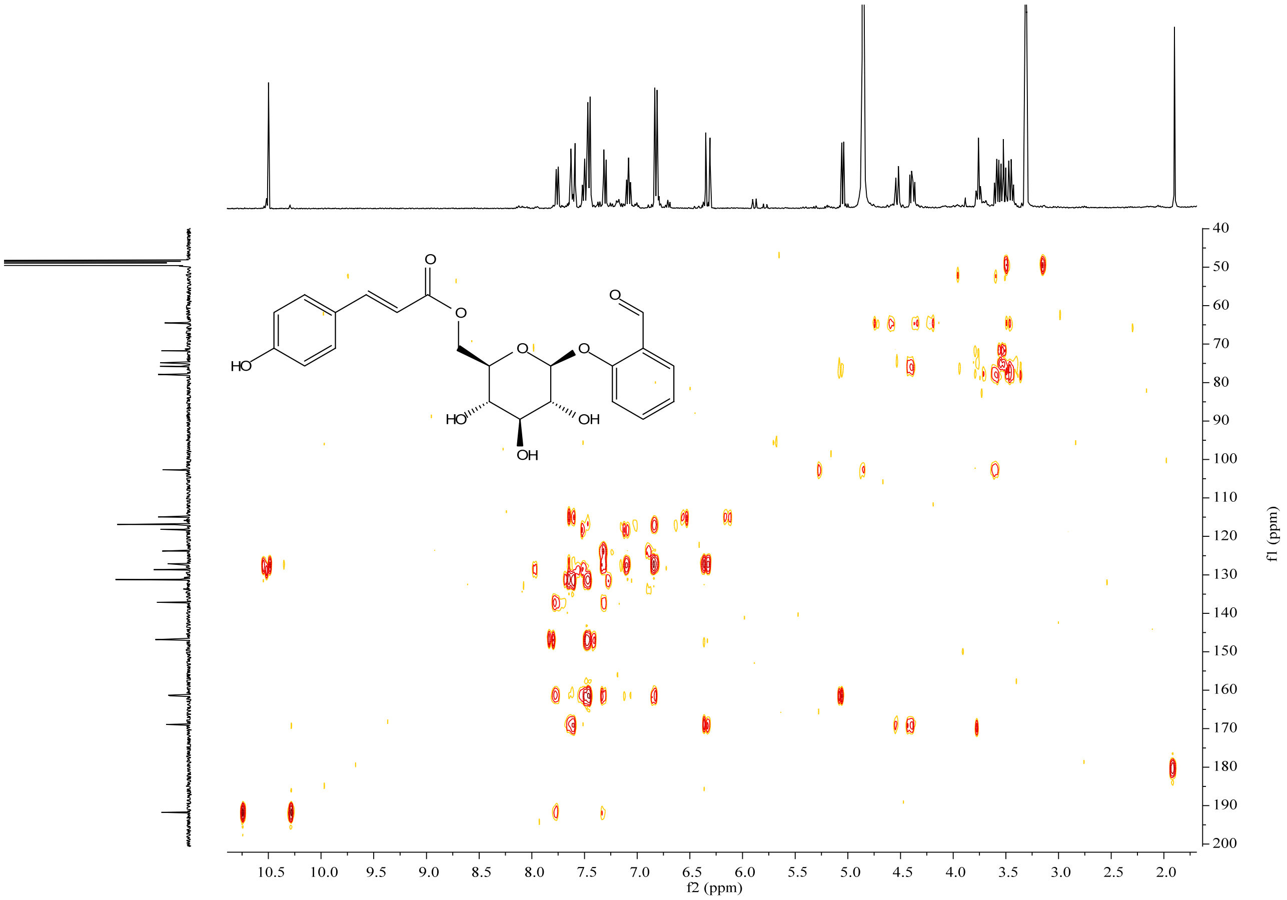

Figure S9. HMBC spectrum of compound **2** in CD_3OD.

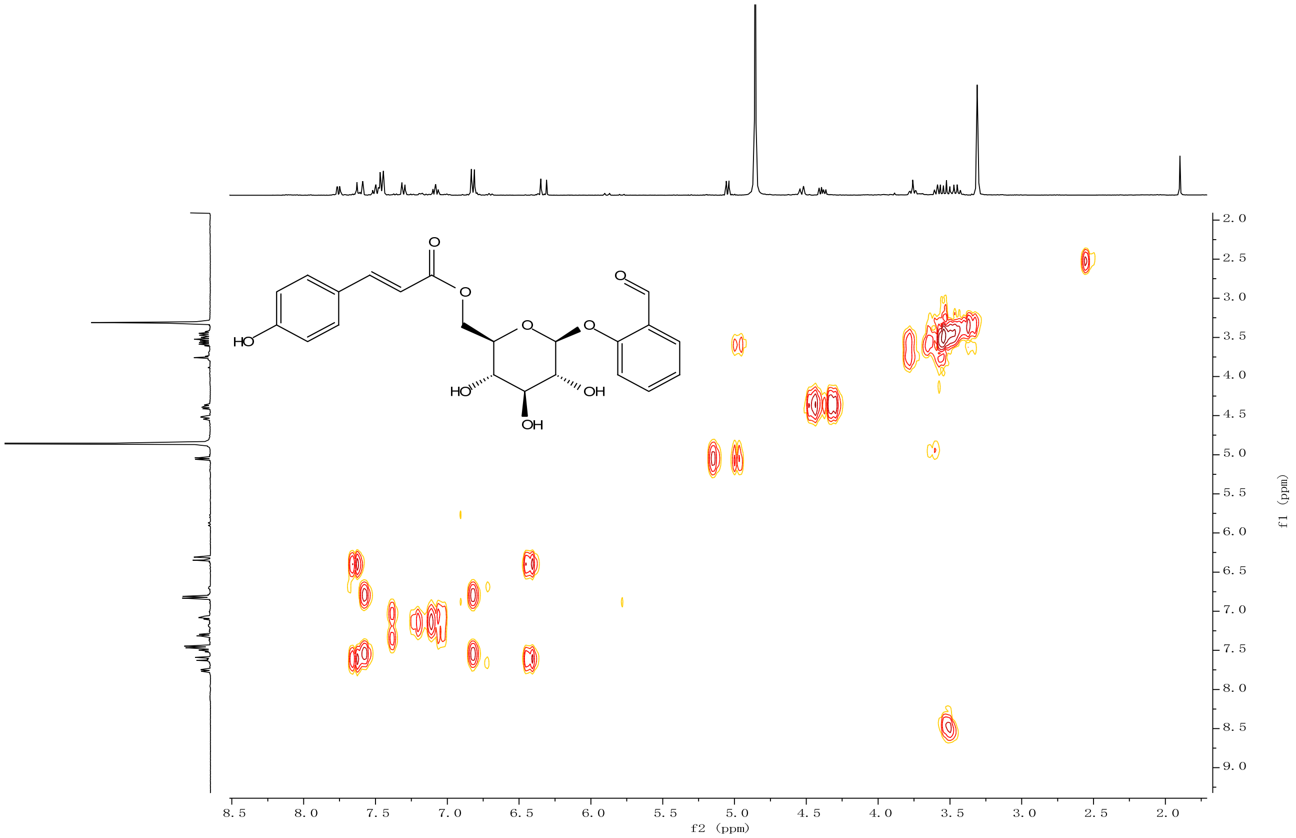

Figure S10. ^1H-^1H COSY spectrum of compound **2** in CD_3OD.

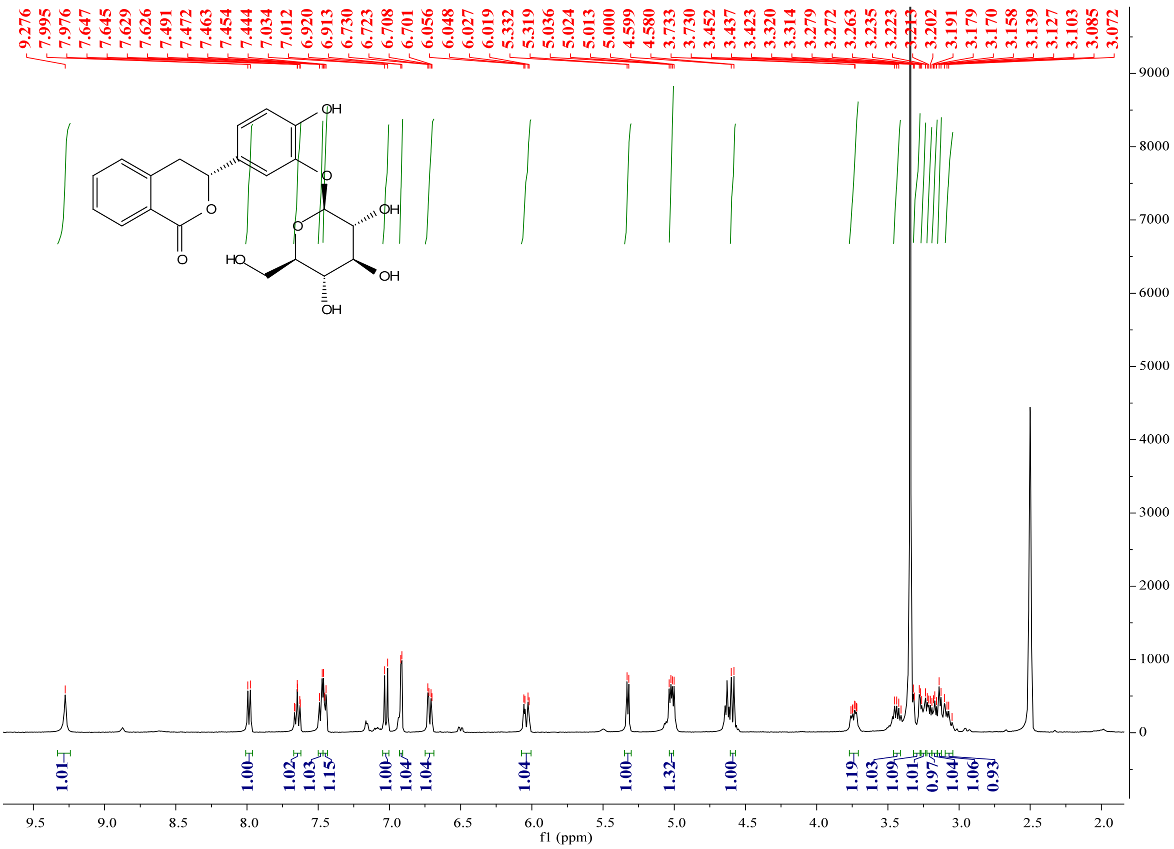

Figure S11. ^{1}H NMR spectrum of compound **3** in DMSO-d_6.

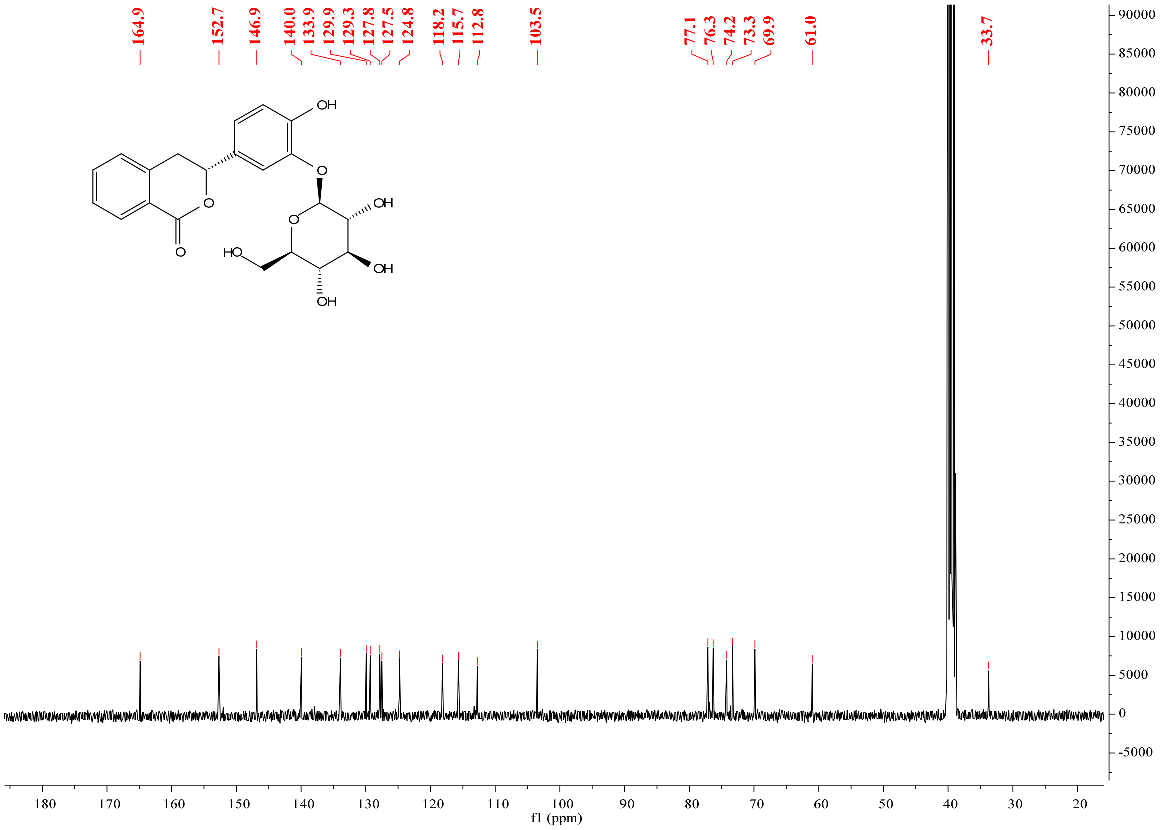

Figure S12. ^{13}C NMR spectrum of compound **3** in DMSO-d_6.

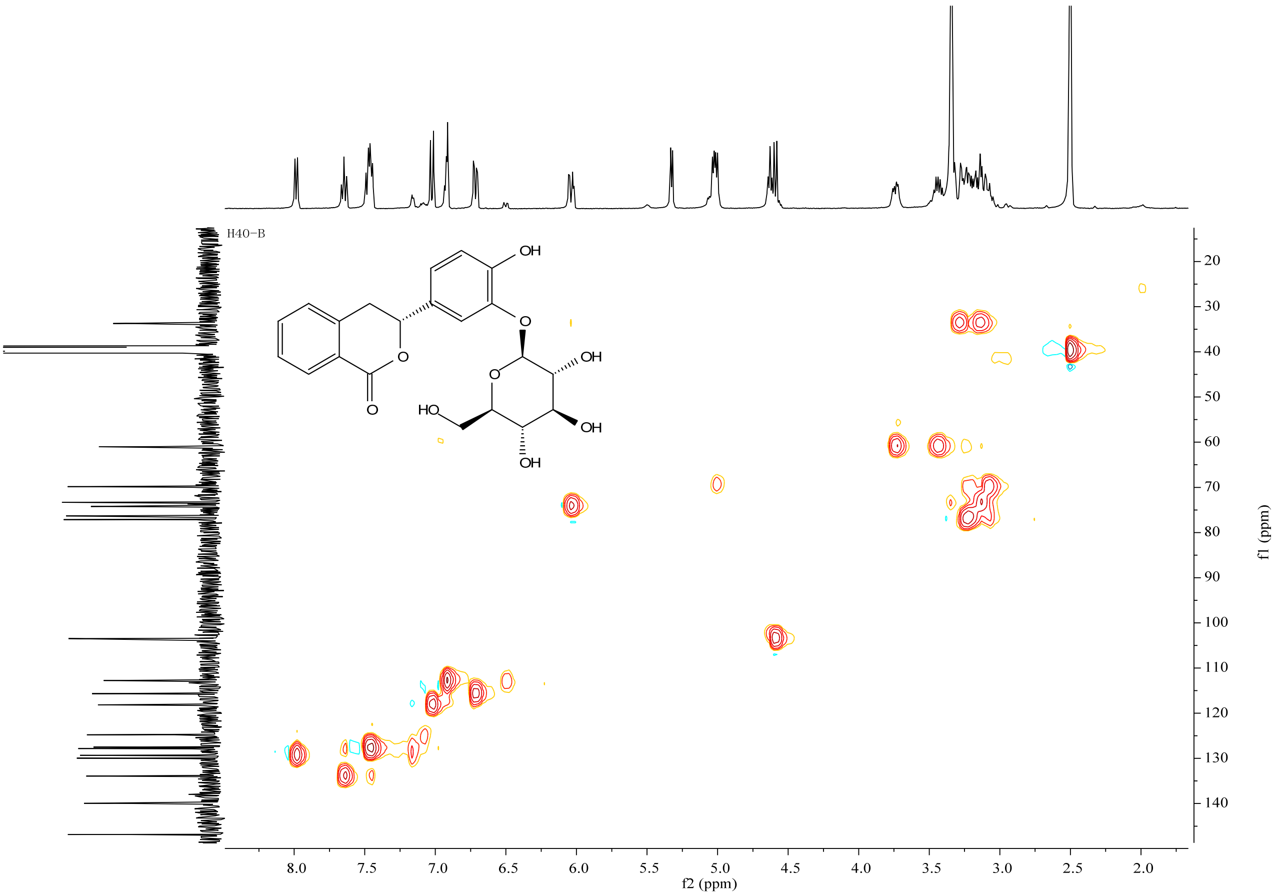

Figure S13. HSQC spectrum of compound **3** in DMSO-d_6.

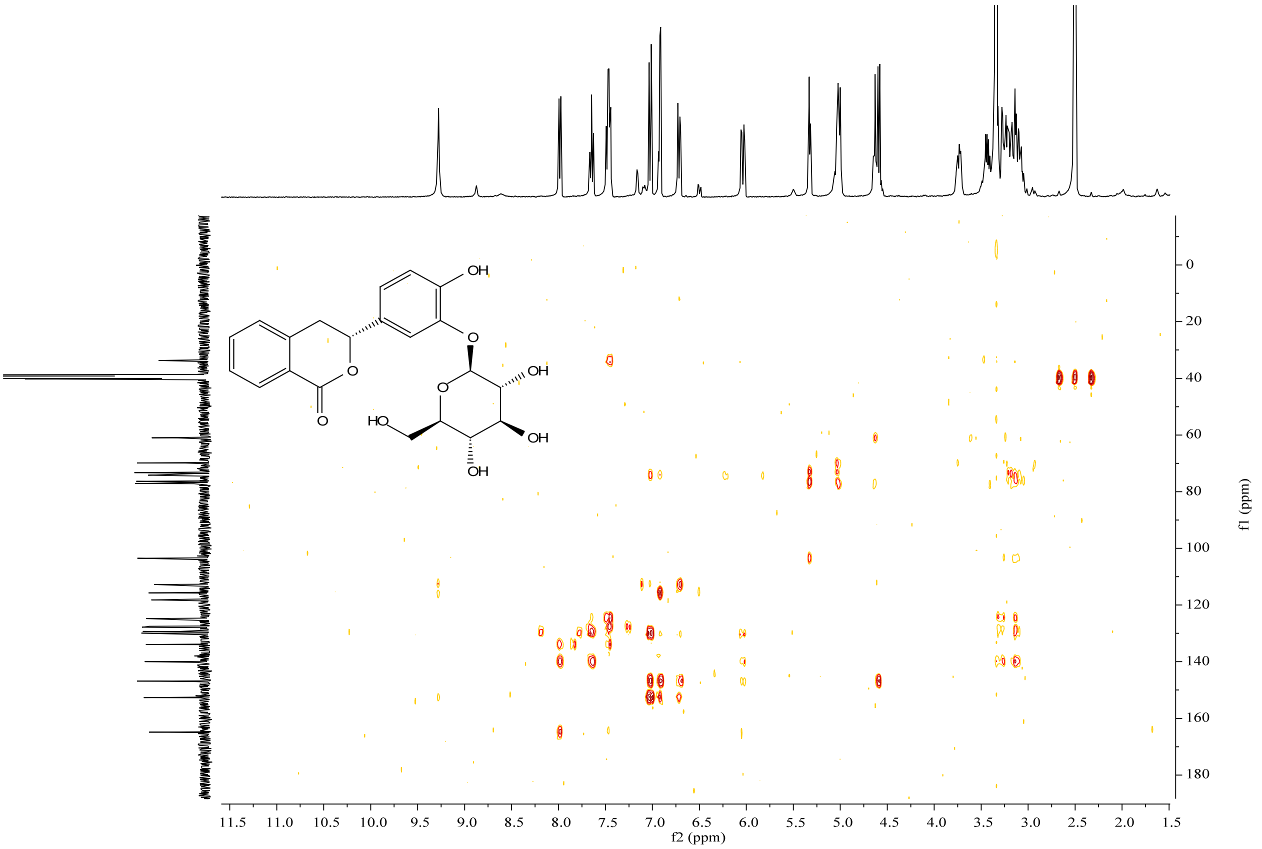

Figure S14. HMBC spectrum of compound **3** in DMSO-d_6.

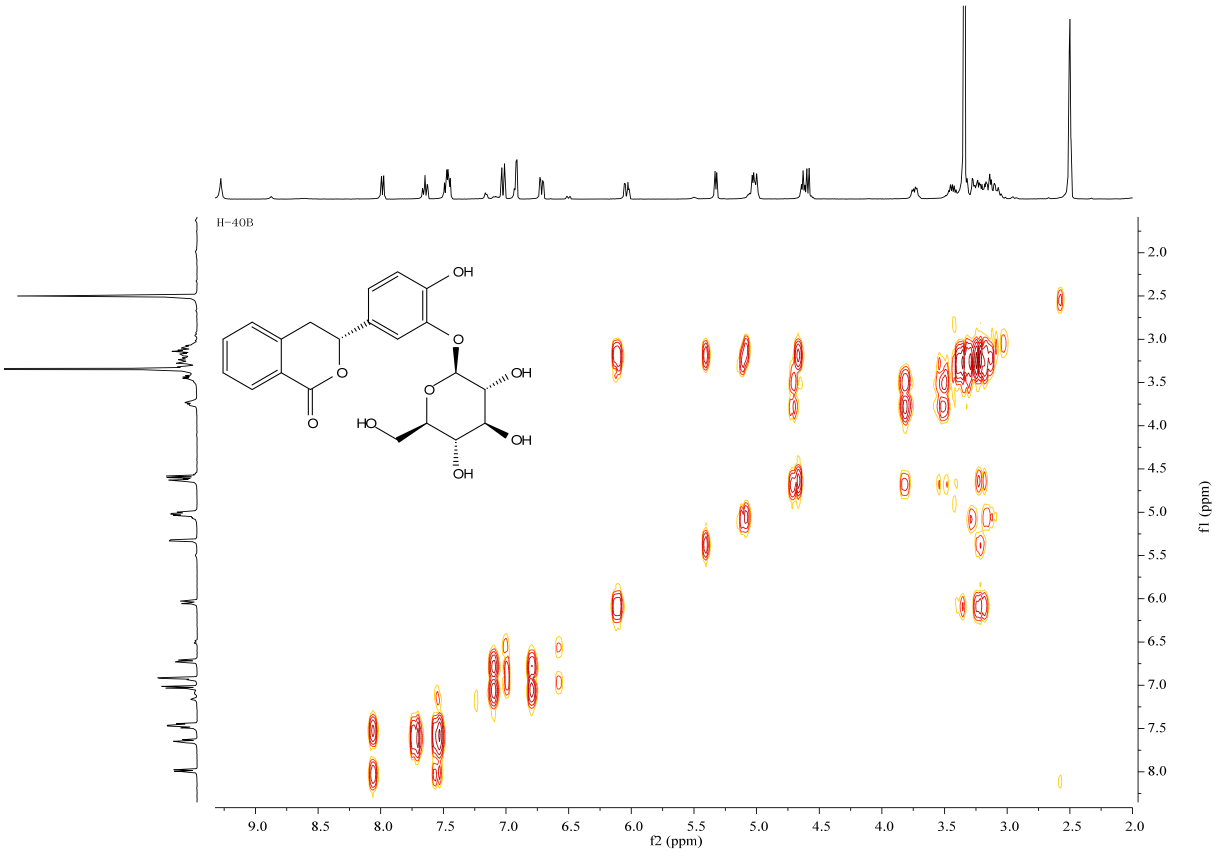

Figure S15. ^{1}H-^{1}H COSY spectrum of compound **3** in DMSO-d_6.

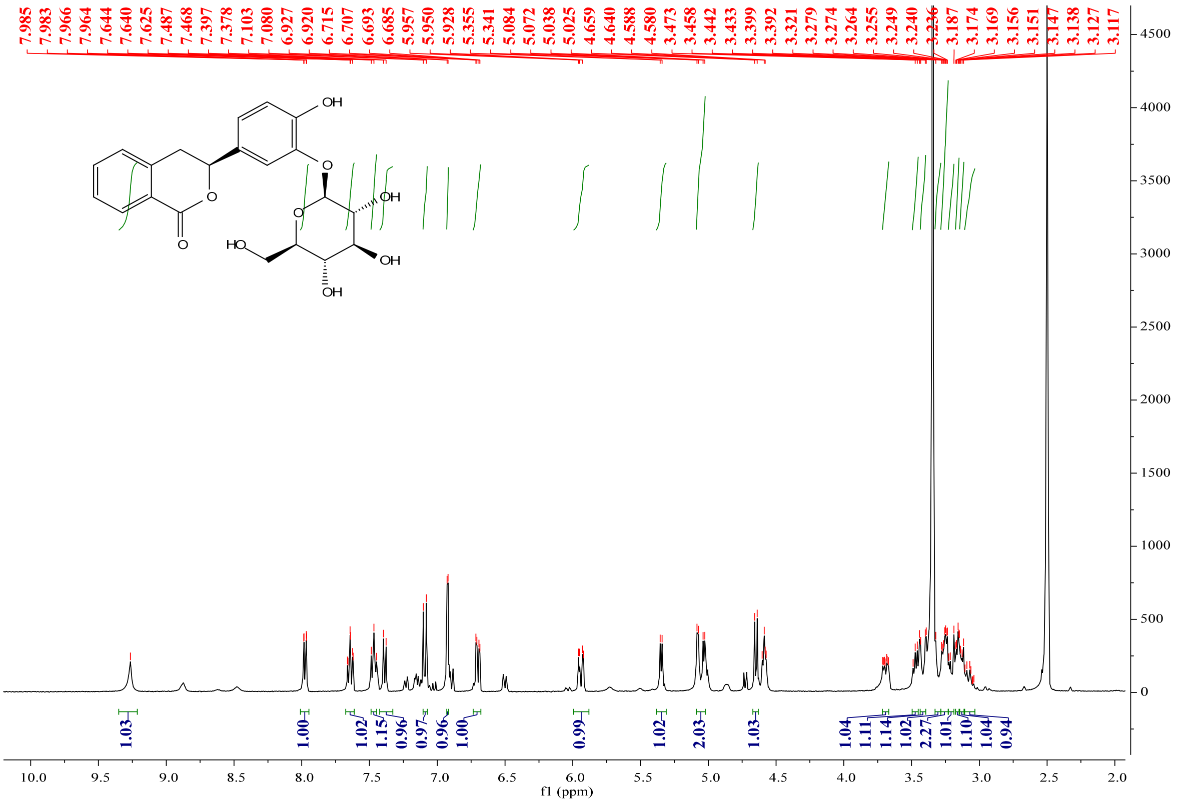

Figure S16. ^{1}H NMR spectrum of compound **4** in DMSO-d_6.

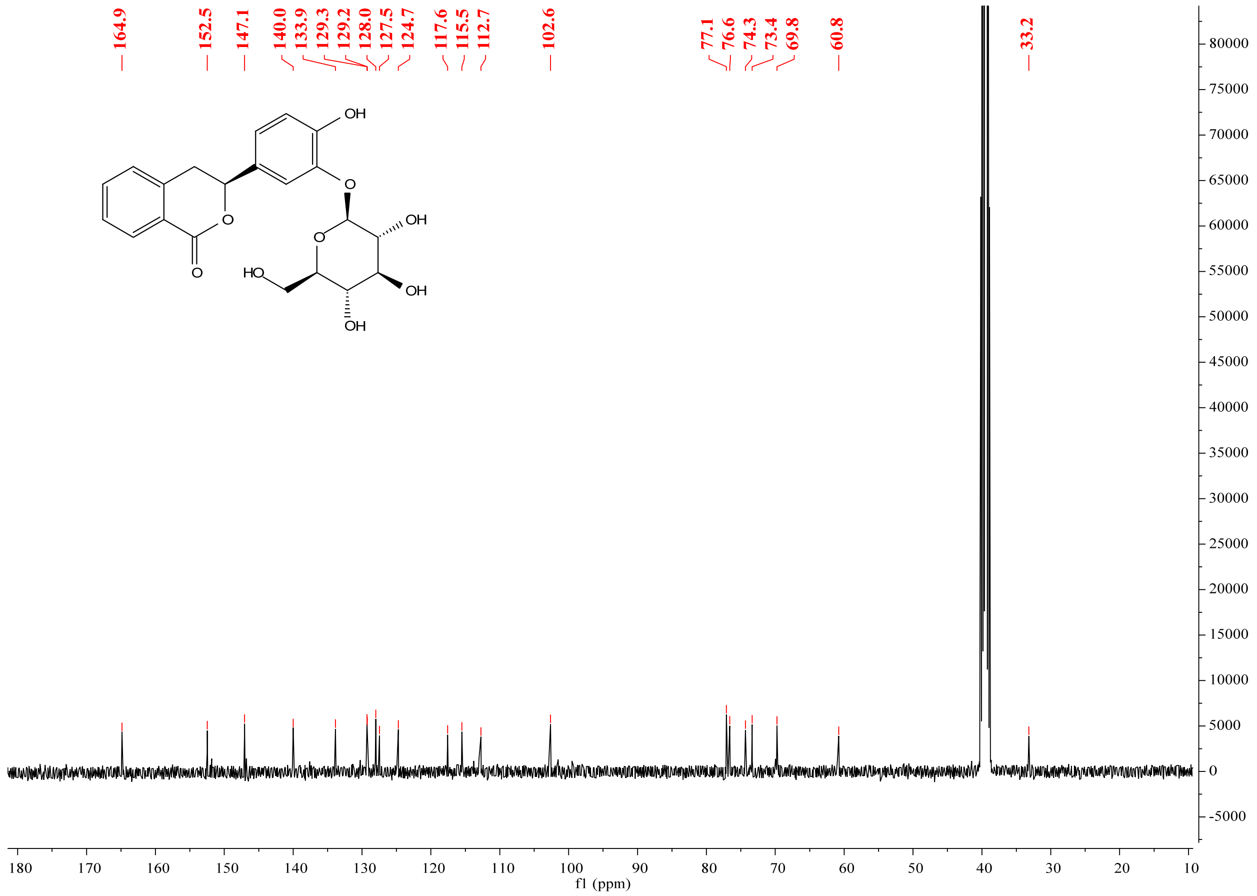

Figure S17. ^{13}C NMR spectrum of compound **4** in DMSO-d_6.

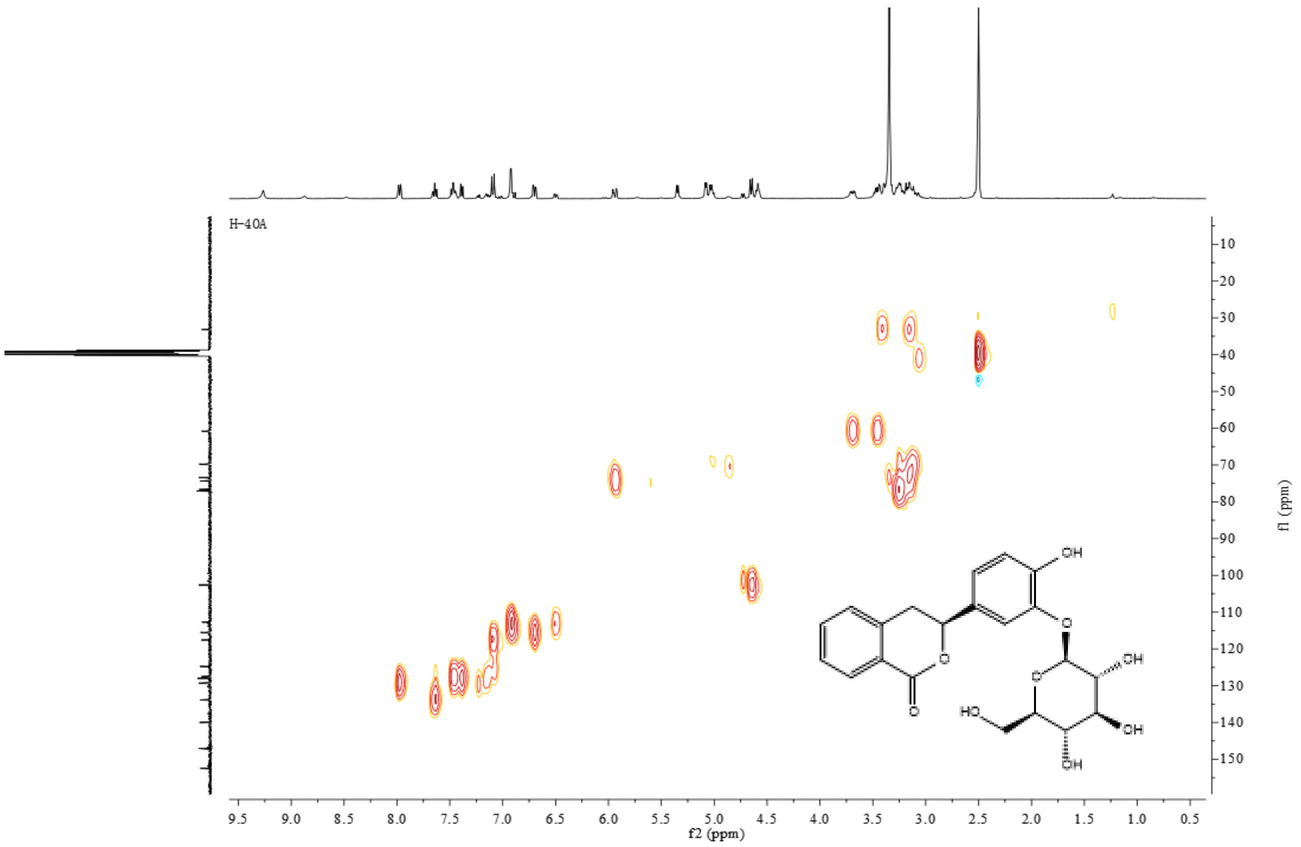

Figure S18. HSQC spectrum of compound **4** in DMSO-d_6.

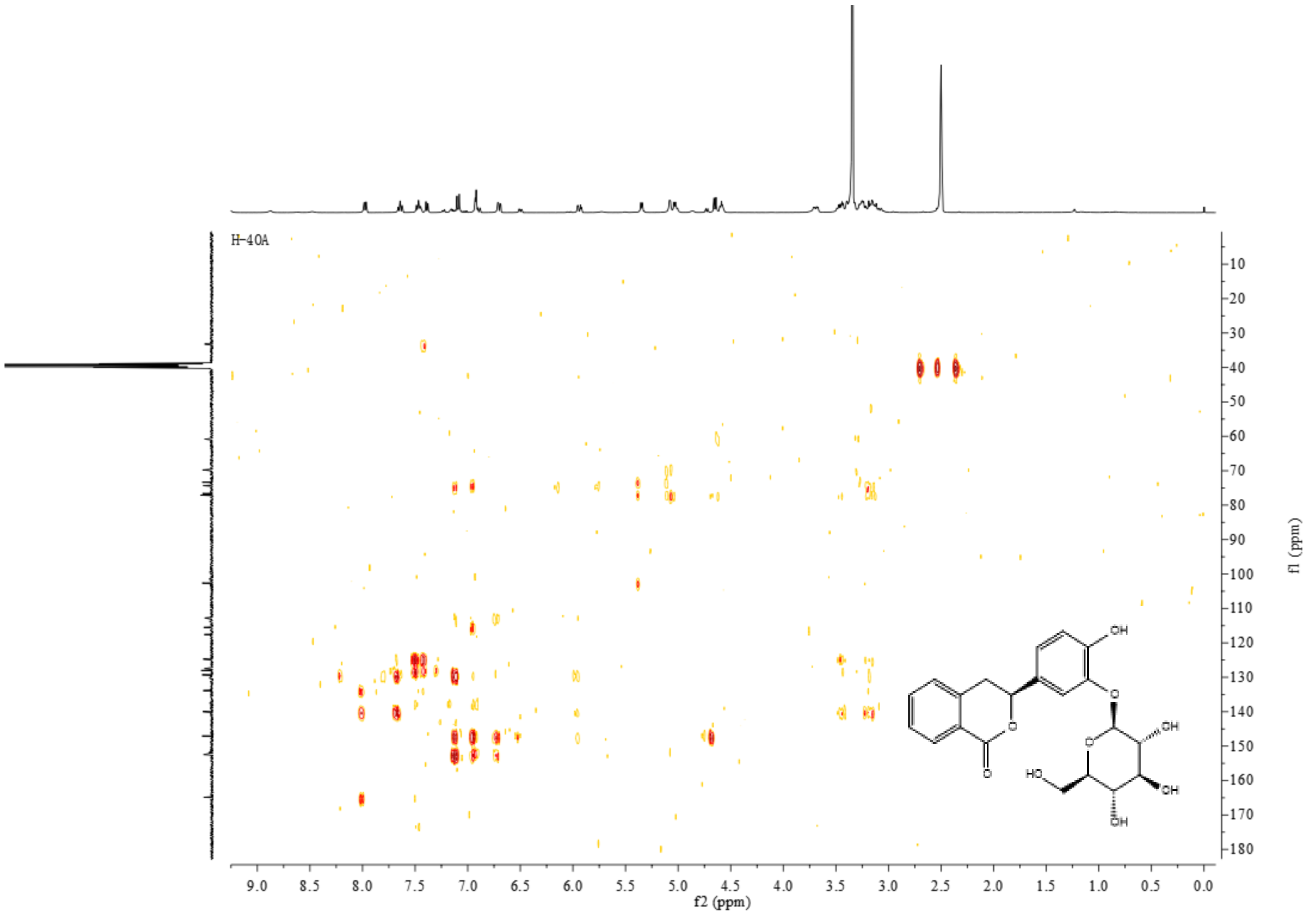

Figure S19. HMBC spectrum of compound **4** in DMSO-d_6.

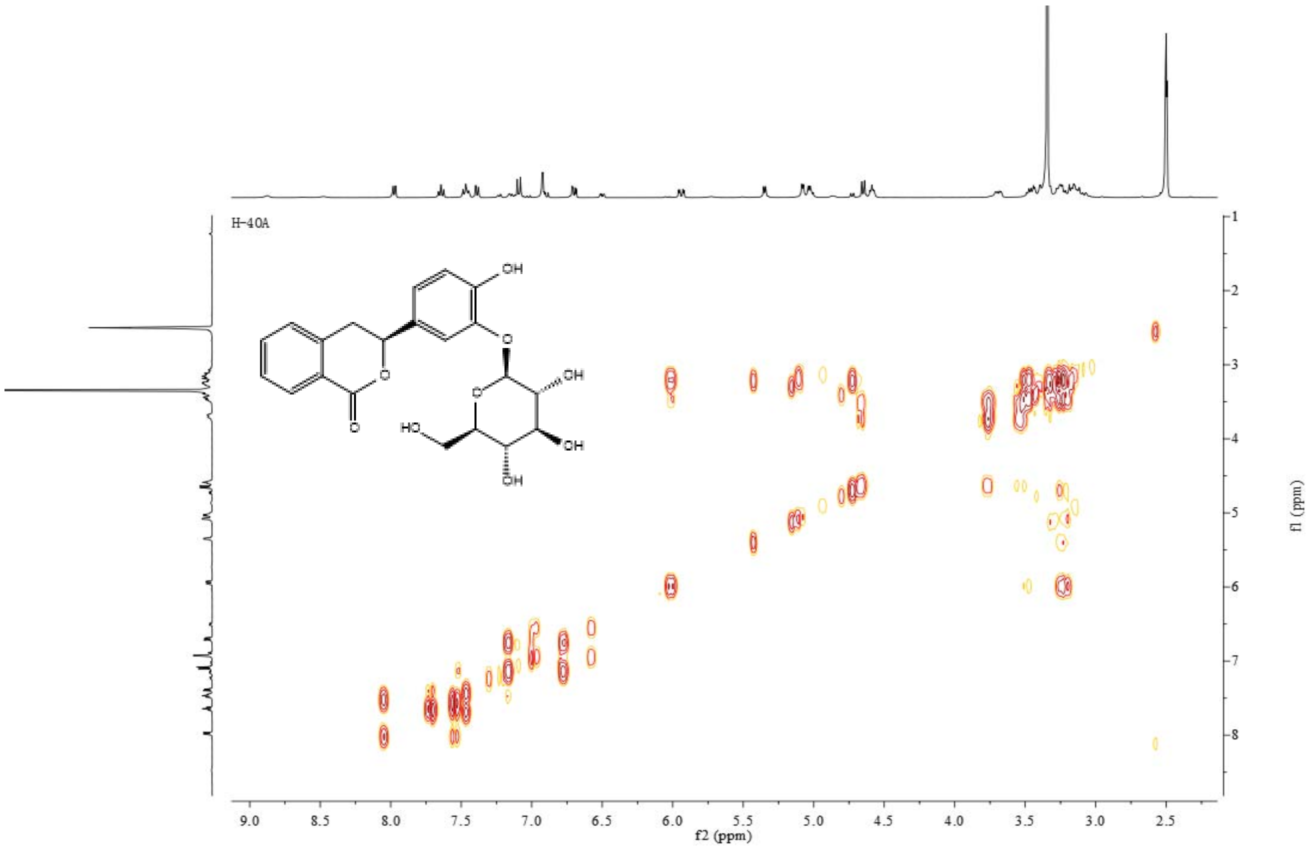

Figure S20. ^1H-^1H COSY spectrum of compound **4** in DMSO-d_6.
